# Language use in depressed and non-depressed mothers and their adolescent offspring

**DOI:** 10.1016/j.jad.2024.08.131

**Published:** 2024-08-24

**Authors:** Laura A. Cariola, Lisa B. Sheeber, Nicholas Allen, Maneesh Bilalpur, Timothy Bird, Saurabh Hinduja, Louis-Philippe Morency, Jeffrey F. Cohn

**Affiliations:** a Clinical and Health Psychology, University of Edinburgh, Edinburgh, UK; b Oregon Research Institute, Eugene, USA; c Department of Psychology, University of Oregon, Eugene, USA; d Intelligent Systems Program, University of Pittsburgh, Pittsburgh, USA; e Department of Management, University of Akron, Ohio, USA; f Language Technologies Institute, Carnegie Mellon University, Pittsburgh, USA; g Department of Psychology, University of Pittsburgh, Deliberate.AI, NY, USA

**Keywords:** Depression, Language behaviour, Dyadic interaction, Depressed mothers, Adolescents, Spoken language, Linguistic analysis

## Abstract

**Background:**

Approximately 10% of mothers experience depression each year, which increases risk for depression in offspring. Currently no research has analysed the linguistic features of depressed mothers and their adolescent offspring during dyadic interactions. We examined the extent to which linguistic features of mothers’ and adolescents’ speech during dyadic interactional tasks could discriminate depressed from non-depressed mothers.

**Methods:**

Computer-assisted linguistic analysis (Linguistic Inquiry and Word Count; LIWC) was applied to transcripts of low-income mother-adolescent dyads (*N* = 151) performing a lab-based problem-solving interaction task. One-way multivariate analyses were conducted to determine linguistic features hypothesized to be related to maternal depressive status that significantly differed in frequency between depressed and non-depressed mothers and higher and lower risk offspring. Logistic regression analyses were performed to classify between dyads belonging to the two groups.

**Results:**

The results showed that linguistic features in mothers’ and their adolescent offsprings’ speech during problem-solving interactions discriminated between maternal depression status. Many, but not all effects, were consistent with those identified in previous research using primarily written text, highlighting the validity and reliability of language behaviour associated with depressive symptomatology across lab-based and natural environmental contexts.

**Limitations:**

Our analyses do not enable to ascertain how mothers’ language behaviour may have influenced their offspring’s communication patterns. We also cannot say how or whether these findings generalize to other contexts or populations.

**Conclusion:**

The findings extend the existing literature on linguistic features of depression by indicating that mothers’ depression is associated with linguistic behaviour during mother-adolescent interaction.

## 1. Introduction

Approximately 10% of mothers experience depression in a given year (Ertel et al., 2011), and maternal depression is associated with significant transdiagnostic risk for offspring (Goodman et al., 2020; Priel et al., 2019). Children with a depressed parent are three to four times more likely to develop depression than children of non-depressed parents (Loechner et al., 2020; Swales et al., 2022). Compelling evidence indicates that both environmental and genetic factors contribute to offspring risk for depression (Gotlib et al., 2020; Natsuaki et al., 2014).

An offspring’s exposure to negative maternal parenting behaviour is an important adverse environmental factor underpinning intergenerational transmission (Weissman et al., 2016; Wolford et al., 2019). Observational research indicated that maternal interactions in daily family environments represent a pathway for the development of psychopathology and behavioural problems in adolescents, including higher rates of depression, greater rates and severity of both internalizing and externalizing symptoms (Jaser et al., 2008). Relative to those of mothers without depression, the parenting practices of depressed mothers are characterized by higher levels of anger, hostility, irritability, negative affect, intrusiveness, criticism, unpredictability, withdrawal and unresponsiveness, as well as lower levels of sensitivity and availability (Fosco and Lydon-Staley, 2019; Lee et al., 2023). Such parenting behaviours adversely impact child outcomes in cognitive, emotional, health, and social domains (Campbell et al., 2004; Ertel et al., 2011; Norcross et al., 2017). These effects are even more pronounced in mothers who experience cumulative stressors (e.g., poverty, separation or divorce, single parenthood, unemployment) (Sartor et al., 2023; Sullivan et al., 2022).

### 1.1. Depression and language behaviour

Computerized language analysis has been used to assess and make inferences about psychological states and functioning in individuals. Computer-assisted approaches to language analysis are based on the assumption that language represents an observable and measurable human behaviour that can be studied in a systematic manner as a data source to develop language-based behavioural prediction models. One of the most prominent and cited computer-assisted methods for language analysis is the Linguistic Inquiry and Word Count (LIWC; Pennebaker et al., 2015), a dictionary-based natural-language programme that measures the frequency of words that have been categorised into semantic and syntactic categories.

The LIWC has been applied to verbal and written data from a range of contexts to identify language associated with depressive symptoms in clinical and non-clinical samples. Self-referential language and language with negative affective content have received the most attention in empirical studies (Jones et al., 2020). Reflecting Pyszczynski and Greenberg (1987) self-awareness theory positing that depressed individuals get stuck in a cycle of repetitive thinking and heightened self-focus in response to a loss of an important source of self-worth, empirical findings have consistently identified a correlation between depression and high frequency use of first-person singular pronoun such as ‘T’, ‘me’, ‘mine’ (Eichstaedt et al., 2018; Ireland and Mehl, 2014; Rude et al., 2004; Sloan, 2005; Tackman et al., 2019; Van den Nest et al., 2019; Zimmermann et al., 2013). Self-referential language during diagnostic interviews conducted in a clinical setting also significantly predicted future depression and was noted as a risk factor or marker in the trajectory of chronic depression (Zimmermann et al., 2017). A meta-analysis by Edwards and Holtzman (2017) identified self-references as a robust and reliable linguistic feature for depression across individuals with varying demographic characteristics, including age and gender.

Consistent with the theory that cognitive schemas in the form of negative beliefs about the self, world, and future (i.e., the Cognitive Triad; Beck, 1969; Beck and Bredemeier, 2016) are a core characteristic of depression, an elevated frequency of negative emotion words, such as those that reflect anxiety (e.g., worried) anger (e.g., hate) and sadness (e.g., crying; De Choudhury et al., 2013; Park et al., 2012; Rude et al., 2004; Tadesse et al., 2019), as well as negation words (e.g., no; Tadesse et al., 2019) have been shown to be associated with depressive symptoms. Conversely, depressed individuals use fewer positive emotion words (e.g., love; Sloan, 2005). This negative focus can create or maintain a sense of helplessness and hopelessness, which then lead to ongoing rumination, obsessive thinking and unhelpful coping behaviours, including avoidant behaviour and social isolation (Kennerley et al., 2017; Moorey, 2010). Negative cognitive styles have also been shown to be associated with stress generation, further contributing to ongoing depression (Liu, 2013; Scott et al., 2007).

Frequent use of cognitive process words (e.g., causation words), potentially as a feature of processing and appraising events and ruminative thinking (Pennebaker et al., 2003), has also been observed to be a linguistic feature of depression (Rodriguez et al., 2010). Similarly, all-or-nothing thinking, as reflected in absolute words (e.g., always) predicts future depression (Al-Mosaiwi and Johnstone, 2018; Eichstaedt et al., 2018). Consistent with the perception that individuals with depression are ‘stuck in the past’ (Holman and Silver, 1998), depression has been associated with an increased use of past-tense words (e.g., did) (Rodriguez et al., 2010; Smirnova et al., 2018; Trifu et al., 2017), whereas the use of present-tense (e.g., do) and future-tense words (e.g., will do) have not been observed consistently in depressed samples (Himmelstein et al., 2018; O’Dea et al., 2017; McNeilly et al., 2023; Rodriguez et al., 2010; Shahane et al., 2023; Tadesse et al., 2019). As would be expected, there is some inconsistency in findings. For example, in a recent study, use of present-focused language was associated with lower average mood, though not depression (McNeilly et al., 2023). The authors suggested that this may be because maintaining a focus on the present may reflect reduced psychological distancing as an effective emotion-regulation strategy (McNeilly et al., 2023; Nook et al., 2022).

Despite the significance of maternal depression for family interactional processes and offspring well-being, there has been very limited study of linguistic patterns in parenting contexts. In one study examining pronoun use in mothers’ verbal narratives, Humphreys et al. (2018) reported that greater self-focus and psychological distancing, as operationalized by more frequent use of “I” and less frequent use of “we” were associated with higher maternal depressive symptoms. In a sample of written tasks, Derella and Milan (2021) found that depressed mothers of children across three developmental age groups, spanning early childhood through adolescence, demonstrated greater self-focus and use of negative words than did mothers not experiencing depression.

A notable feature of the above-described literature is that it is based largely on written language either as shared in social media or in response to textual prompts as part of language tasks. There is considerably less data about linguistic features of depressed persons’ spoken language in everyday interactional contexts. One study examining depressed mothers’ speech within structured observations of parent-child interactions during home visits (Goodlett et al., 2017) assessed maternal positive-emotion word use (e.g., love) as an indicator of maternal positivity and a potential buffer between maternal depressive symptoms and child internalizing problems in a vulnerable sample (i.e., low-income, ethnic minority families raising young children in an impoverished urban context). Their findings indicated that mothers’ use of positive emotion words during an attachment-script assessment partially attenuated the link between maternal depressive symptoms and at-risk young children’s internalizing problems. However, there has not been consideration of the possible impact of gender-specific socialisation in language use within the interactional speech context between the depressed parents and their children. In contrast, studies in nonselected samples of mothers and their children during interactional speech have explored gender-specific socialisation in expressive language (Aznar and Tenenbaum, 2013, 2020). For example, earlier studies have shown that in talking with daughters, relative to sons, mothers discuss a greater variety of emotions as well as discussing negative emotions more frequently (Fivush et al., 2000; Kuebli et al., 1995). Gender differences in emotion talk were identified with girls by age 6 using more emotion language, particularly more positive emotion expressions, compared to boys who use more negative emotion expressions (Chaplin and Aldao, 2013; Fivush et al., 2000; Kuebli et al., 1995). These studies focused predominantly on emotion talk, and little is known about broader gender-specific language differences in the mother-child interactional context.

### 1.2. Current study

Despite the influence of depressive conditions on family relations and offspring outcomes, only limited research examined linguistic behaviour in face-to-face interactions within the parenting context. To address this gap in the empirical literature, the primary goal of our study was to examine the extent to which language variance is attributable to maternal depression in the speech of mothers and their adolescent offspring focusing on linguistic features reflecting self-referential pronouns, temporal and emotion language, and secondarily, the extent to which maternal depressive status could be predicted based on their own and their offspring’s language. We did not, however, explore interactional patterns within the dyadic mother-child task. Our approach is novel, as to our knowledge, no research has analysed the linguistic behaviour of depressed mothers and their adolescent offspring. Our examination of adolescent speech was predicated on evidence that maternal depression is a strong risk factor for offspring depression, as described earlier. As linguistic behaviour appears to be a predictor, and not merely a marker of depression (Derella and Milan, 2021; Zimmermann et al., 2017), observing similar patterns in offspring would provide preliminary support for the hypothesis that maternal language may present one mechanism of environmental risk—that is, that children may learn maladaptive language patterns via dynamic interactions they have within their familial environment.

We applied LIWC analysis to the transcripts of recorded lab-based mother-adolescent problem-solving interactions (PSI), focusing on a set of salient linguistic features that have been consistently associated with depressive symptomatology as well as those associated with poorer quality interpersonal relationships. Based on the described above, our confirmatory hypothesis predicted that depressed mothers and their higher-risk adolescent offspring would use significantly more frequently (a) first-person singular pronouns, (b) negation words, (c) negative emotion words (i.e., anger, sadness, anxiety), (d) cognitive process words, (e) past-tense words, (f) present-tense words, and (g) future-tense words, but (h) significantly fewer positive emotion words, compared to non-depressed mothers and their lower-risk adolescent offspring. Given the interpersonal context of the PSI task of this study, (i) we also predicted that depressed mothers and their higher-risk adolescent offspring would use significantly lower frequency of first-person plural pronouns and higher frequency of second-person pronouns, indicating increased psychological distancing and lower quality (e.g., lower cohesion, higher conflict) in the parent-child relationship (Simmons et al., 2005) compared to non-depressed mothers and their lower-risk adolescent offspring.

Given existing evidence that identified gender-specific socialisation of emotion talk in general samples of mothers with their offspring, our exploratory hypotheses focused on the main and interactional effects of offspring’s gender on mothers’ linguistic features, and the main and interaction effects of offspring’s risk status and gender on each linguistic feature.

Finally, we conducted logistic regression analyses to determine whether the identified linguistic features that significantly differ between mothers in the depressed vs. non-depressed group, and between their respective offspring would accurately classify between dyads with depressed and non-depressed mothers.

## 2. Method

### 2.1. Data and sample characteristics

The data were collected as part of a study examining proximal social-affective predictors of parenting behaviour in depressed and non-depressed, low-income mothers of young adolescents (*N* = 180 dyads; Nelson et al., 2021a, 2021b). Two groups of women were recruited: 1) a depressed group, selected for current elevated depressive symptoms (PHQ-8 > 10; Kroenke et al., 2009) and a history of treatment for depression; 2) a non-depressed group, selected for no or mild levels of current depressive symptoms (PHQ-8 < 8), no history of treatment for depression, and no current (i.e., past month) mental health treatment for any disorder. Low-income was operationalized as Medicaid eligibility. We focused on a low-income population because such disadvantage is associated with more adverse parenting behaviour, a primary focus of the broader study (Barajas-Gonzalez and Brooks-Gunn, 2014; Kavanaugh et al., 2018). Exclusion criteria for mothers and adolescents included current diagnosis of psychosis, other illness, or cognitive impairment that would interfere with participation.

Data from 151 mother and adolescent dyads, who had given informed consent and assent, respectively, for data sharing were included in the analyses. Mothers completed the PHQ-9 (Kroenke et al., 2001) at the time of the assessment. Mothers in the depressed group (*N* = 74) had a mean PHQ-9 of 12.20 (SD = 5.93, range = 2–25) and mothers in the non-depressed group (*N* = 77) had a mean PHQ-9 of 2.62 (SD = 2.77, range = 0–12). The two groups of women (depressed vs non-depressed) and their adolescent offspring (higher-risk vs lower-risk) did not differ on age or race/ethnicity. Demographic data for mothers and adolescents are presented in Table 1.

### 2.2. Recruitment

The majority of participants were recruited through the organization that administered the Oregon Health Plan (Medicaid) in the county where data were collected. The remaining participants were recruited through online advertisements and screened to ensure Medicaid eligibility. Mothers and adolescents were compensated for their participation, including travel expenses.

### 2.3. Assessment procedure

#### Family-interaction assessment:

Mother-adolescent dyads completed a 15-minute, lab-based Problem-Solving Interaction (PSI), in which they were asked to discuss and try to resolve two issues of conflict selected from an updated Issues Checklist (Prinz et al., 1979). The checklist was updated so that language and topics would be relatable and understandable to study participants. The PSI task has been shown to elicit a full range of affective behaviour, while differentially eliciting negative affect (Nelson et al., 2017, 2021a, 2021b; Schwartz et al., 2014). The interactions were video-recorded. Topics selected were those with the highest mean frequency by intensity ratings across mother and adolescent reports on a 5-point Likert scale ranging from ‘Calm’ (1), ‘A little angry’ (3) to ‘Angry’ (5). Ratings of the intensity of discussions when topics were discussed in the home significantly differed between groups, *t(134)* = −*4.74*, *p* < *.001 (15 missing values)*, with non-depressed dyads rating topics as less intense *(M* = *2.04, SD* = *0.73*) compared to the depressed dyads *(M* = *2.68, SD* = *0.84)*, indicating that, on average, discussions were not rated as particularly charged by participants in either group.

### 2.4. Measures

#### 2.4.1. Diagnostic measures

To characterize the sample and ensure that participants in the non-depressed group did not meet criteria for current or past depressive disorder, mothers completed an abbreviated Structured Clinical Interview, non-patient version (SCID-NP; First et al., 2024). Overall interrater reliability, based on 20% of the data was kappa = 0.80.

#### 2.4.2. Self-report questionnaires

To assess the presence and severity of depression, mothers completed the Patient Health Questionnaire-9 (PHQ-9) (Kroenke et al., 2001).

#### 2.4.3. Linguistic measure

Audio recordings collected during the PSI task were manually segmented and transcribed, by native English speakers. We defined utterances as continuous spoken activity with <300 ms of silence. Each utterance was characterized with a start and stop time. Filler words such as “mhm”, “uhh”, “err” were also transcribed and punctuation was preserved. Transcriptions were reviewed to ensure accuracy. During the transcription process, the speaker of each utterance was identified along with the spoken content. In case of overlapping speech, both speakers were identified, and content was transcribed. We removed words that were not discernible to the annotators and corrected any misspelling manually. Subsequently, the English version of the LIWC (Pennebaker et al., 2015) was administered to all transcripts.

The LIWC is a dictionary-based natural language program that measures the percentage of words that have been categorised into semantic and syntactic categories. The LIWC comprises approximately 4500 words and word stems that are categorised into 80 semantic categories and subcategories. The semantic categories are organized within three overarching categories, including ‘Linguistic Processes’ (e.g., personal pronouns, articles, verbs), ‘Psychological Processes’ (e.g., social processes, affective processes, cognitive processes), and ‘Personal Concerns’ (e.g., work, leisure, death). The LIWC is hierarchically organized—for example, all sadness words belong to the sub-category ‘negative emotions’ which forms part of ‘affect words’ category. In this study, we initially selected the following linguistic categories: personal pronouns (i.e., first-person singular pronouns, first-person plural pronouns, second-person pronouns), negation words, positive emotions, negative emotions and its sub-categories anger, anxiety, and sadness, cognitive processes, and temporal processes (i.e., past-tense, present-tense, future-tense).

### 2.5. Data analysis

Statistical analyses were conducted using IBM SPSS Statistics for Windows, Version 26.0 (IBM Crop, Armonk, NY). Descriptive statistics were computed for the linguistic features (Table 2). All linguistic features were standardised by converting the percentages into z-scores (Newman et al., 2003). We conducted collinearity diagnostics of mothers’ and offspring’ linguistic features and excluded ‘negative emotions’ from further analysis due to tolerance below 0.2 and variance inflation factor and tolerance (VIF) above 7 in both matrixes (Field, 2018).

To determine linguistic features that significantly differed in frequency between depressed and non-depressed mothers and between lower-risk and higher-risk offspring, we conducted a series of one-way multivariate analyses (MANOVAs) with the selected linguistic features as the dependent variables and depression status as the independent variable. The multivariate model for mothers included offspring’s gender as an independent variable. The multivariate model for offspring included gender as an additional independent variable and age as a covariate.

Logistic regression analyses were conducted with mothers’ depression status as the dependent variable, with mothers’ (Model 1) and offsprings’ (Model 2) linguistic features as predictor variables, respectively. Only linguistic features demonstrating significant differences as identified in the one-way MANOVAs (*p* < .05) were maintained for forced entry logistic regression analyses. To control for the effect of mothers’ language use on offsprings’ language use, a forward stepwise approach with Wald statistics was used for Model 3 due to the large number of independent variables that included all mothers’ linguistic categories and all offspring’s linguistic markers. Statistical significance for the regression tests was set at *p* < .05. ‘Non-depressed dyads’ were coded as ‘0’ and ‘depressed dyads’ were coded as ‘1’. Depression was coded as within-subjects and between-subjects correlation matrixes are provided as supplementary material.

### 2.6. Ethics

As part of the original study, mothers and adolescents provided informed consent and assent, respectively, prior to participation, including to the data sharing that enabled the current project. The initial study as well as sharing of the data for secondary analysis was approved and monitored by Oregon Research Institute Review Board (IRB). Secondary-data analysis was approved by the University of Edinburgh’s ethics committee.

## 3. Results

### 3.1. Assessing main effects of group on language use

The descriptive statistics of the linguistic features by mothers’ depression status and offspring risk status, respectively, and univariate main effects of one-way MANOVAs can be seen in Table 2.

The results revealed significant multivariate effect differences between depressed and non-depressed mothers on language features, *F (12,136) = 3.076, p = .001, Wilks’ lambda* Λ = *0.79, ηp^2^ = 0.21*. Univariate results revealed linguistic features that distinguished between depressed mothers using *first-person singular pronouns, second-person singular pronouns, negation words, present-tense words* more frequently but *past-tense words* less frequently than non-depressed mothers. The main effect for mothers’ linguistic features between offspring’s gender, *p = .30*, and the interaction between mothers’ depression status and offspring’s gender on mothers’ linguistic features, *p = .36*, were not significant.

There were significant multivariate effects for differences in linguistic features between higher and lower-risk offspring, *F(12,135) = 2.518, p = .005, Wilks’ lambda* Λ = 0.82, *ηp^2^ = 0.18*. Individual univariate results revealed linguistic features that distinguished higher-risk offspring using *second-person pronouns, anger words, sadness words, present-tense words* more frequently but *future-tense words* less frequently than lower-risk offspring.

Main effects were also identified for offspring’ gender on language, *F (12, 135) = 2.183, p = .016, Wilks’ lambda* Λ = *0.84*, *ηp^2^ = 0.16*, explaining differences between groups at a multivariate level. Individual univariate results revealed that daughters used more *anger words (M = 0.73, SD = 0.79, p = .04)* than sons *(M = 0.48, SD = 0.58)*, and more *future-tense words (M = 2.21, SD = 1.04, p = .001)* than sons *(M = 1.71, SD = 0.87)*. No main effects were identified for age, *p = .13*, or the interaction between offspring’s risk status and gender, *p = .71*, on language use.

### 3.2. Classifying mothers’ depression status based on mother and offspring language use

Based on mothers’ linguistic categories, Model 1 was significant, *χ^2^ = 29.39, p = .001, R^2^N = 0.24*, indicating that an increased frequency of *negation words* and reduced frequency of *past-tense words* were significant predictors of maternal group, correctly classifying 68.9% of cases (Table 3).

Based on adolescent’s linguistic categories, Model 2 was significant, *χ^2^ = 25.64, p = .001, R^2^N = 0.21*, indicating that an increased frequency of *sadness words*, along with a reduced frequency of *future-tense words* were significant predictors of mothers’ group status, correctly classifying 69.5% of cases (Table 4).

Controlling for the effect of mother’s language use on adolescent’s language behaviour, Model 3 was significant, *χ^2^ = 35.14, p = .001, R^2^N = 0.28*, correctly classifying 68.9% of cases. No changes were identified in mothers’ and offsprings’ linguistic categories as predictors of depression status compared to Model 2, indicating that mothers’ language use in the dyadic PSI task did not appear to have a substantial effect on adolescents’ language use (Table 5).

## 4. Discussion

The aim of this study was to examine linguistic dimensions in the speech of depressed mothers and their adolescent offspring during an interactional context. To date research has mainly focussed on adults rather than children, and there has been no research to our knowledge into linguistic qualities of depressed persons’ speech in face-to-face interactions, and very limited examination of language in parenting contexts, despite the influence of depressive conditions on family relations and offspring outcomes. The semi-naturalistic conversational data set used in this study has the advantage that it includes a clinical population of women of lower socioeconomic status. This contrasts with the more frequent use of large social media data sets, often utilised in computerized language studies, which have been criticised for lacking construct validity with the identification and prediction of mental health status in data sets due to issues around defining how mental health conditions are measured and operationalized (Chancellor and De Choudhury, 2020).

The findings of the multivariate analyses were partially consistent with those of previous research with a focus on depressed people generally in finding that mothers in the depressed group used more first-person singular pronouns, which has been interpreted as an indicator of heightened self-focus and rumination (e.g., Eichstaedt et al., 2018; Ireland and Mehl, 2014; Rude et al., 2004; Sloan, 2005; Tackman et al., 2019; Van den Nest et al., 2019; Zimmermann et al., 2013). We also observed that mothers in the depressed group used more second-person pronouns. In the context of problem-solving interactions it seems likely that use of the pronoun ‘you’ may be indicative of blaming statements (“you always…”) or suggestions that the other person change their behaviour (“if you would only…”), such that higher use is associated with lower interactional quality and relationship satisfaction (Georgiou et al., 2011; Simmons et al., 2005). It should be noted that in contexts other than dispute resolution, second-person pronouns have been shown to function more positively (Packard and Berger, 2020).

Contrary to hypotheses and past research that identified increased frequency of negative emotions words (i.e., anxiety, anger, sadness words) with depression (Park et al., 2012; Rude et al., 2004; Tadesse et al., 2019; Tausczik and Pennebaker, 2010), our findings did not show a significant group difference in frequency of negative emotion words. However, there was a significant difference of negations which also significantly contributed to the logistic regression model distinguishing group status. Whereas negative emotion words are explicit expressions of negative mood states, negations implicitly communicate negative schemas associated with depressed mood states through the use of negative terms (e.g., no, not, never) (Fine, 2008; Tadesse et al., 2019). Also, no significant difference emerged for positive emotion words between depressed and non-depressed mothers. These findings are surprising given robust evidence of reduced positivity and harsh parenting behaviour in depressed mothers (Goodman et al., 2020). It should be noted that emotion word use, though overlapping with these constructs, is distinct from observed affect, criticism, or warmth and acceptance, which are the variables often captured in the maternal depression literature (Gotlib et al., 2020). It seems likely that the findings showing no significant differences for negative or positive emotion words may reflect the difference in the context of language between this research using spoken language in a dyadic context and previous studies focussing on written language. However, it is also notable that these findings are consistent with those that emerged in a recent study of linguistic features in a social messaging study of adolescents (McNeilly et al., 2023).

Though both ruminating about the past and having a negative outlook regarding the future are characteristic of depression, temporal words, such as present, past, and future words, have received only limited attention in empirical research. Our findings indicate that temporal processes are relevant linguistic features, with depressed mothers using more present-tense words and fewer past-tense words. Reduced frequencies of past-tense words in depressed relative to non-depressed mothers also contributed to the prediction model. This finding is inconsistent with some previous literature in which depression was associated with greater use of past-tense words (Rodriguez et al., 2010; Smirnova et al., 2018; Trifu et al., 2017), consistent with being ‘stuck in the past’. Increased use of present-tense words relative to past- and future-tense, however, has been found in some prior research, and as noted earlier, may reflect less effective use of psychological distancing as an emotion regulation strategy (McNeilly et al., 2023: Nook et al., 2017, 2020, 2022).

Psychological distancing enables persons to take a step back from emotionally charged situations. Evidence suggests that greater use of present-tense language, particularly in combination with more frequent use of first-person singular pronouns, may reflect reduced psychological distancing (Nook et al., 2017). Lesser use of psychological distancing is associated with less effective emotion regulation (Nook et al., 2017, 2020), as well as higher levels of perceived stress and depressive symptoms, and lower levels of emotional well-being in adults (Shahane et al., 2023). Notably, these effects have been shown in naturalistic as well as lab-based data sets. For example, linguistic indices of increased psychological distancing over time have been shown to be associated with reduction in psychological distress, following analysis of a large corpus of text-based psychotherapy conversations (Nook et al., 2022). Similarly, in a sample of adolescents, McNeilly et al. (2023) found that participants who used more present-focused words in smart-phone communication (i.e., lower psychological distancing) evidenced lower moods (if also not more depressive symptoms) than those who used fewer present-focused words. It is worth noting here, that as described by McNeilly et al. (2023), these findings may seem counter-intuitive given the emphasis on mindfulness-based emotion regulation approaches on present-focused attention; however, as the authors noted, the use of present-focus words in social communication may be a form of secondary processing rather than an indication of mindful awareness.

Linguistic features in the speech of adolescent offspring were also associated with maternal depressive status. There were significant differences in the frequency of negative emotion words, including anger and sadness words in the speech of higher-risk adolescents, with sadness words contributing to the prediction model. Adolescent offspring of depressed mothers also demonstrated higher rates of second-person pronouns, which as discussed, may reflect a greater sense that the other person is responsible for their difficulties.

Heightened levels of negative emotion words in offspring of depressed mothers could be a reflection of difficulties understanding and regulating emotions (Denham et al., 2007; Hooper et al., 2015; Scharfe, 2000), which have been associated with adverse and less supportive parenting behaviour and exposure to depressed mothers’ own negative-emotion expression and emotion regulation difficulties, including anger and feelings of frustration (Cohn et al., 1990; Silk et al., 2006). While neither mothers in the depressed nor the non-depressed groups spoke differently with their daughters and sons, the findings revealed that overall daughters used more anger words compared to sons. Offspring of depressed mothers also used more present-tense words but fewer future-tense words, and across groups, daughters used more future-tense words than did sons. This finding regarding temporal language in the offspring was of interest considering a lack of research on temporal words in adolescents; however previous literature with a focus on adults described inconsistent findings of the use of past-tense and future-tense words in depression (Rodriguez et al., 2010; Smirnova et al., 2018; Trifu et al., 2017).

Several possible explanations of the linguistic differences in speech of higher-risk and lower-risk adolescents exist. First, the linguistic features of offspring of depressed women may reflect their own risk for depressive conditions, whether from genetic and environmental risk factors they share with their mothers (Hawrilenko et al., 2019; Lussier et al., 2021; Sallis et al., 2017). Second, consistent with social learning and family system theories, linguistic patterns may be learned through socialisation experiences that occur through communication with a depressed parent. Alternatively, because the adolescent language data were derived during interactions with their mothers, it is also possible that differences emerge as a function of proximal maternal influences in the dialogue. That is, differential linguistic characteristics in higher-risk offspring may have emerged because the adolescents were interacting with a depressed person. This is an important possibility given the well-established differences in communication patterns of depressed and non-depressed mothers in interaction with their children (Goodman et al., 2020). To address this possibility, we conducted a logistic regression analysis of offspring’s language predicting mothers’ depression status, controlling for mothers’ language use. Results indicated that maternal language did not appear to be a primary contributor to offspring’s language use. Yet, in the absence of speech samples gathered in other dyadic interactions, such as during communication with peers, it is not possible to determine the generalizability of between-group differences in offspring language across contexts.

An additional consideration is that, given the well-established finding that maternal depression is associated with elevations of conflict during parent-child interactions (Fosco and Lydon-Staley, 2019; Lee et al., 2023), the linguistic differences observed in this study could be due to differences in the intensity of the discussions in dyads in which the mothers are depressed in comparison with those in which they are not. Ratings of the intensity of discussions on the lab-selected topics—when discussed at home—revealed that participants in the depressed group rated intensity to be slightly higher than did those in the non-depressed group. Notably, ratings within both groups were between 2 and 3 on a scale in which 1 was ‘calm’, 3 was ‘a little angry’, and 5 was ‘angry’, indicating that, on average, discussions were not rated as particularly charged by participants in either group. As the discussions took place in the home, we do not have intensity ratings for interactions that took place in the lab prior to the PSI task. Due to the time delay between the home-based discussions, lab-based intensity ratings and the PSI task, as well as families’ discretion to change topics, it was perceived as unjustified to make predictions regarding possible effects of intensity and topics on between-group differences in language use. Nonetheless, it is possible that differences in the felt intensity of the discussions and topics may have been related to differences in language use in the PSI task.

Overall, we found that many, but not all effects, were comparable to those that emerged in different modalities of expression used in previous research, including studies that examined social media posts (De Choudhury et al., 2013; Park et al., 2012; Tadesse et al., 2019) and lab-based essays (Rude et al., 2004). This overlap suggests the external validity and ecological validity of language behaviour associated with depressive symptomatology across contexts.

Notably, the findings of this study may have clinical implications with regards to the potential influence of linguistic factors on mother and adolescent well-being. For example, research examining depressed persons’ language use in therapy transcripts has found associations between improvements in depression and an increased use of discrepancy words and positive emotion words, but fewer past focus and negation words (Huston et al., 2019; Van der Zanden et al., 2014). Increased frequency of self-references was not associated with depressive symptoms at baseline, but was at discharge, and was a significant predictor of depressive symptoms at 8 months follow-up in a prospective longitudinal study (Zimmermann et al., 2017). Additionally, data from longitudinal studies of expressive writing have indicated that only the frequency of non-fluencies was significantly associated with symptoms of depression at the group-level, and no significant correlations were identified between linguistic features and changes in symptoms at the individual level in blog content written over a 36-week period (O’Dea et al., 2017). Together, these findings suggest that various linguistic features have been associated with depression at baseline and outcome in both therapeutic and expressive writing interventions. However, inconsistent empirical evidence across these different interventions indicates that further research is required to obtain a more detailed insight into language as a vehicle to assess depression at baseline and outcome, and its mechanisms in the reduction of symptoms as part of the therapeutic process.

Our findings add to those of prior research in also raising the possibility that mothers’ language use during conversations with their children may have implications for offspring adjustment. This opens up the possibility that changes in language use as a therapeutic outcome in depressed individuals may not only have implications for the wellbeing of the individual, but also that of their family members. Consistent with this possibility is a body of research which indicates that the quality of caregiver- and whole-family-child interactions predicts later development of children’s knowledge, regulation skills and expression of emotions (Cooper et al., 2023; Paley and Hajal, 2022; Reschke et al., 2024). As such, the findings of this study may matter from both intervention and etiological perspectives.

## 5. Limitations and future directions

As this was the first study examining linguistic features of depressed mothers and their adolescent offspring during dyadic interactions, it will be important to examine the extent to which findings replicate in future studies, especially those in more naturalistic settings (Nelson and Allen, 2018). As the present sample was fairly homogeneous with regard to race, ethnicity, and income, it will be important to examine linguistic features of speech in more diverse samples; this is particularly important in that disruptions in parenting associated with depression may be experienced to a greater degree by ethnic-minority and economically disadvantaged mothers (Barajas-Gonzalez and Brooks-Gunn, 2014; Watson et al., 2019).

Relatedly, as noted earlier, we do not know from this data, how mothers’ language behaviour within or outside the lab-based interaction may have influenced their offspring’s’ communication. To overcome this limitation, future research could explore the use of computer-assisted linguistic analysis of language behaviour associated with depressive symptoms in both mothers and their offspring using a time-series approach to explore dynamic interactive processes. We also do not know whether adolescent language patterns relate more broadly to interactional or relationship factors within the home, or shared environmental conditions, which provide important future directions to explore mechanisms (e.g., spillover and compensatory effects; Nelson et al., 2009) to explain how parental psychopathology may influence emotion socialisation and parent-child interaction within family systems.

While using dyadic data of mother-child engaging in a problem-solving task, this study focused primarily on exploring language differences between depressed vs non-depressed mothers, and higher-risk and lower-risk offspring. Future research will examine interactional patterns and effects within the dyadic mother-child interactions, such as interactive components and sequential patters of language use that are common within and between dyads of depressed and non-depressed groups.

## 6. Conclusion

This study provided insights into linguistic dimensions that differentiate depressed mothers and their adolescent offspring from their peers in families without a depressed mother during mother-adolescent problem-solving interactions. Pending replication and extension, as described above, these novel findings may have important implications for early intervention and clinical practice in families experiencing maternal depression.

## Supplementary Material

Appendix

## Figures and Tables

**Table 1:** Mothers’ and Adolescents’ demographic characteristics by group.

	Depressed Mothers		Non-Depressed Mothers		Higher-Risk Adolescents		Lower-Risk Adolescents	
	N	M (SD)	N	M (SD)	N	M (SD)	N	M (SD)
Age	74	40.32 (6.49)	77	40.07 (6.27)	74	12.86 (1.19)	77	12.87 (1.23)
Gender identity								
Female	74		77		34		31	
Race								
American Indian/Alaska Native	1		3		0		1	
Asian	1		0		0		0	
Native Hawaiian/Pacific islander	0		2		0		1	
Black or African American	0		1		0		1	
White or Caucasian	65		63		60		58	
Multiple Races	7		6		14		14	
No response/unknown	0		2		0		2	
Ethnicity								
Latino or Spanish descent	4		9		11		19	
No Latino or Spanish descent	70		68		63		58	

N = number of participants; M = mean; SD = standard deviation.

**Table 2:** Mothers’ and Adolescents’ frequencies of linguistic features and univariate main effects by group.

	Depressed Mothers		Non-Depressed Mothers				Higher-Risk Adolescents		Lower-Risk Adolescents			
	N = 74		N = 77				N = 74		N = 77			
	M	SD	M	SD	*F*	*p*	M	SD	M	SD	*F*	*p*
Personal pronouns												
1st person singular pronouns	4.56	1.97	3.95	1.49	5.31	0.02*	8.87	2.62	8.31	2.64	1.70	0.20
1st person plural pronouns	1.64	1.04	1.59	0.85	0.13	0.72	1.35	1.21	1.31	1.11	0.00	0.96
2nd person pronouns	7.76	1.86	7.08	2.05	4.71	0.03*	2.51	1.84	1.86	1.26	5.98	0.02*
Negations	3.22	1.11	2.54	0.85	17.29	0.01**	4.47	2.17	4.09	1.98	1.13	0.20
Affect												
Positive emotions	3.76	1.18	4.15	1.69	2.40	0.12	4.15	2.54	4.06	2.36	0.02	0.90
Negative emotions	1.59	0.90	1.28	0.84	n/a		1.95	1.21	1.33	0.85	n/a	
Anger	0.62	0.66	0.43	0.53	2.74	0.10	0.73	0.77	0.45	0.58	4.71	0.03*
Anxiety	0.17	0.21	0.18	0.23	0.00	0.96	0.14	0.32	0.11	0.23	0.92	0.34
Sadness	0.25	0.27	0.22	0.23	0.26	0.61	0.42	0.55	0.21	0.23	9.03	0.01*
Cognition	15.31	2.25	14.79	2.23	1.98	0.16	14.33	3.22	13.63	2.71	2.62	0.11
Time												
Past tense	2.26	1.07	2.75	1.40	5.48	0.02*	2.78	1.79	2.91	1.87	0.20	0.66
Present tense	18.31	2.40	17.11	2.12	11.83	0.01**	16.45	3.10	15.37	3.07	4.26	0.04*
Future tense	1.83	0.71	2.06	0.82	2.54	0.11	1.71	0.95	2.13	0.96	8.82	0.01*

N = number of participants; M = mean; SD = standard deviation; F = main effect.

* p < .05.

** p < .01.

**Table 3:** Binary logistic regression analysis of mothers’ linguistics features associated with maternal depressed group.

Factors	β	SE	Wald	Adjusted OR	95% CI		p
					Lower	Upper	
1st person singular pronouns	0.14	0.12	1.43	1.15	0.91	1.46	0.23
2nd person pronouns	0.14	0.10	2.03	1.15	0.95	1.40	0.15
Negations	0.66	0.22	9.00	1.94	0.1.26	2.98	0.01**
Present-tense	0.03	0.10	5.42	1.03	0.84	1.26	0.79
Past-tense	−0.42	0.18	2.62	0.66	0.46	0.94	0.02*

β = unstandardized regression coefficient; SE = standard error; OR = odds ratio; CI = confidence interval. Non-depressed dyads = 0, Depressed dyads = 1.

* p < .05.

** p < .01.

**Table 4:** Binary logistic regression analysis of adolescent offspring’s linguistics features associated with maternal depressed group.

Factors	β	SE	Wald	Adjusted OR	95% CI		p
					Lower	Upper	
2nd person pronouns	0.18	0.12	2.01	1.19	0.94	1.52	0.15
Anger	0.39	0.28	1.90	1.47	0.85	2.55	0.17
Sadness	1.22	0.54	5.15	3.39	1.18	9.70	0.02*
Present-tense	0.07	0.06	1.14	1.07	0.95	1.20	0.29
Future-tense	−0.52	0.20	6.78	0.60	0.41	0.88	0.01**

β = unstandardized regression coefficient; SE = standard error; OR = odds ratio; CI = confidence interval. Non-depressed dyads = 0, Depressed dyads = 1.

* p < .05.

** p < .01.

**Table 5:** Binary logistic regression analysis of mothers’ and offsprings’ linguistics features associated with maternal depressed group.

Factors	β	SE	Wald	Adjusted OR	95% CI		p
					Lower	Upper	
Mother_Negations	0.67	0.21	10.23	1.96	1.30	2.97	0.01**
Mother_Past-tense	−0.43	0.16	7.21	0.65	0.48	0.89	0.01**
Offspring_Sadness	1.07	0.54	3.98	2.91	1.02	8.32	0.05*
Offspring_Future-tense	−0.46	0.20	4.98	0.64	0.43	0.95	0.03*

β = unstandardized regression coefficient; SE = standard error; OR = odds ratio; CI = confidence interval. Non-depressed dyads = 0, Depressed dyads = 1.

* p < .05.

** p < .01.
