# Supplementary material for: Language use in depressed and non-depressed mothers and their adolescent offspring
Source: J Affect Disord. Author manuscript; Available in PMC 2024 Dec 18. (PMC11654823; doi:10.1016/j.jad.2024.08.131)
Supplement: Appendix [file NIHMS2019692-supplement-Appendix.pdf]

Supplementary Material

Correlation Table of Linguistic Features in Non-Depressed Mothers

Variable	1	2	3	4	5	6	7	8	9	10	11
1. 1st person singular pronouns	-										
2. 1st person plural pronouns	-.06	-									
3. 2nd person pronouns	-.12	-.42**	-								
4. Negations	.19	-.28**	.13	-							
5. Positive emotions	-.09	-.19	-.25**	.16	-						
6. Anger	-.04	-.12	.21	.25*	.03	-					
7. Anxiety	.09	-.05	.04	-.16	.04	-.02	-				
8. Sadness	.19	.05	.00	.19	.11	.05	.10	-			
9. Cognition	-.00	-.03	.18	-.05	-.18	.10	.07	-.24*	-		
10. Past tense	-.06	-.22	.08	.13	.15	.04	.09	.19	-.23*	-	
11. Present tense	.40**	.10	-.01	.14	-.29*	.09	.02	-.00	.13	-.51**	-
12. Future tense	.05	.20	-.22	-.04	-.12	-.15	-.16	-.05	-.09	-.23*	.25*

Note: * p < .05, ** p < .01

Correlation Table of Linguistic Features in Depressed Mothers

Variable	1	2	3	4	5	6	7	8	9	10	11
1. 1st person singular pronouns	-										
2. 1st person plural pronouns	-.40	-									
3. 2nd person pronouns	-.30*	-.43**	-								
4. Negations	.27*	-.19	.27*	-							
5. Positive emotions	-.02	.09	-.09	-.16	-						
6. Anger	-.02	.11	.10	.24*	-.09	-					
7. Anxiety	-.06	-.08	-.02	-.06	.01	.05	-				
8. Sadness	.31**	.08	-.16	.38**	.00	-.15	-.02	-			
9. Cognition	.21	.17	.07	.16	-.22	-.13	-.11	.30*	-		
10. Past tense	.20	.05	-.15	.17	-.12	-.22	.15	.18	-.08	-	
11. Present tense	.32**	.00	.19	.37**	-.13	.11	.03	.03	.33**	-.26*	-
12. Future tense	-.13	.01	.09	-.15	-.10	.04	-.06	-.34**	-.07	-.17	.24*

Note: * p < .05, ** p < .01

Correlation Table of Linguistic Features in Lower-Risk Adolescents

Variable	1	2	3	4	5	6	7	8	9	10	11
1. 1st person singular pronouns											
2. 1st person plural pronouns	-.54**	-									
3. 2nd person pronouns	.16	-.09	-								
4. Negations	.35**	-.22	.14	-							
5. Positive emotions	.19	-.02	.19	.31**	-						
6. Anger	.17	.12	.36**	.20	.01	-					
7. Anxiety	-.17	-.09	.15	-.12	.10	-.04	-				
8. Sadness	-.14	.06	.29**	-.17	.25*	-.01	.08	-			
9. Cognition	-.08	.03	-.17	.24*	-.09	.11	-.11	-.29*	-		
10. Past tense	.07	-.15	.17	-.07	.04	-.10	.12	.23*	-.38**	-	
11. Present tense	.21	.14	.13	.26*	.04	-.09	.25*	-.01	.30**	-.42**	-
12. Future tense	-.38**	.57**	-.05	-.16	.00	-.02	.03	.17	-.18	-.17	.03

Note: * p < .05, ** p < .01

Correlation Table of Linguistic Features in Higher-Risk Adolescents

Variable	1	2	3	4	5	6	7	8	9	10	11
1. 1st person singular pronouns	-										
2. 1st person plural pronouns	-.53**	-									
3. 2nd person pronouns	.13	-.28*	-								
4. Negations	.19	.04	.05	-							
5. Positive emotions	-.11	-.03	.15	.06	-						
6. Anger	-.06	-.24*	.21	-.03	-.20	-					
7. Anxiety	.10	-.02	.21	-.10	.08	.01	-				
8. Sadness	.16	-.00	-.06	.04	-.01	-.05	.12	-			
9. Cognition	.28*	-.15	-.09	.24*	.06	-.30**	.07	.13	-		
10. Past tense	-.10	.02	.12	.01	.01	-.13	.16	.09	-.15	-	
11. Present tense	.52**	-.21	.17	.25*	-.16	-.02	.12	.28*	.34**	-.18	-
12. Future tense	-.05	.10	.03	-.28	-.10	.01	.11	-.01	-.08	.12	-.03

Note: * p < .05, ** p < .01

Correlation Table of Linguistic Features between Non-Depressed Mothers and Low-Risk Adolescents

Variable	Offspring_1st person singular pronouns	Offspring_1st person plural pronouns	Offspring_2nd person pronouns	Offspring_Negations	Offspring_ Positive emotions	Offspring_Anger	Offspring_Anxiety	Offspring_Sadness	Offspring_Cognition	Offspring_Past tense	Offspring_Present tense	Offspring_Future Tense
Mother_1st person singular pronouns	.15	.03	.17	.16	.08	-.03	.09	.05	.05	-.15	.29*	-.15
Mother_1st person plural pronouns	-.21	.24*	-.26*	-.18	-.17	-.07	-.07	.11	-.12	-.11	-.06	.13
Mother_2nd person pronouns	.33**	-.31**	.18	.24*	-.00	-.10	.05	-.24	.05	-.03	_-.09	-.12
Mother_Negations	.28*	.05	.20	.24*	.24*	-.18	.20	.09	-.06	.14	.19	-.11
Mother_Positive emotions	-.28*	.10	.22	-.10	.30**	.27*	-.02	.26*	.02	.21	-.03	-.10
Mother_Anger	-.01	.16	.30**	.12	.13	.65**	-.09	.22	-.12	.08	.10	.10
Mother_Anxiety	-.06	.06	.08	-.00	-.05	.23*	.24*	-.16	-.06	.07	-.04	-.02
Mother_Sadness	-.01	.10	.01	.09	.19	-.15	.02	.18	-.09	.12	.06	.03
Mother_Cognition	-.07	.16	-.07	-.04	-.11	.18	.13	-.33**	.42**	-.36**	.14	.16
Mother_Past tense	.15	-.18	.21	.04	.02	.08	.07	-.01	-.10	.46**	-.11	-.24*
Mother_Present tense	.14	.04	.05	.06	.04	-.19	.13	-.09	-.04	.34**	.34**	-.03
Mother_Future tense	.02	.09	-.18	.04	-.11	.05	.04	-.07	-.04	.03	.03	.03

Note: * p < .05, ** p < .01

Correlation Table of Linguistic Features between Depressed Mothers and Higher-Risk Adolescents

Variable	Offspring_1st person singular pronouns	Offspring_1st person plural pronouns	Offspring_2nd person pronouns	Offspring_Negations	Offspring_ Positive emotions	Offspring_Anger	Offspring_Anxiety	Offspring_Sadness	Offspring_Cognition	Offspring_Past tense	Offspring_Present tense	Offspring_Future Tense
Mother_1st person singular pronouns	.27*	-.04	.45**	.14	-.12	.02	.18	.21	.10	.15	.30*	.06
Mother_1st person plural pronouns	-.08	.38**	-.11	.15	-.01	.02	.04	-.01	.21	-.09	.15	.00
Mother_2nd person pronouns	.20	-.30**	.07	.12	-.07	.00	.07	.06	-.22	.06	-.03	-.26*
Mother_Negations	.12	-.23*	.22	.20	-.05	-.13	.38**	.26*	.09	.30*	.13	-.12
Mother_Positive emotions	-.24*	.06	.06	-.10	.27*	-.09	-.10	-.11	-.08	-.06	-.15	.01
Mother_Anger	.02	-.12	.06	-.04	.02	.52**	.05	.03	-.03	.20	-.06	-.01
Mother_Anxiety	-.02	-.08	-.04	-.10	.03	-.05	.26*	-.01	-.01	-19	.03	-.01
Mother_Sadness	-.02	-.01	.04	-.08	-.17	-.07	.27*	.27*	.06	.10	-.01	.03
Mother_Cognition	.13	.08	.11	.26**	-.03	-.02	-.01	.12	.37**	-.15	.25*	-.15
Mother_Past tense	-.02	.03	.22	-.02	-.07	-12	.20	.05	.14	.44**	-.03	.21
Mother_Present tense	.30*	-.15	.19	.34**	-.09	.04	.23	.19	.14	.02	.43**	-.20
Mother_Future tense	.18	-.16	-.09	.17	.06	-.07	-.10	-.01	.04	.08	.06	.15

Note: * $p < .05$, ** $p < .01$
